# Inhibition of IGF-1-Mediated Cellular Migration and Invasion by Migracin A in Ovarian Clear Cell Carcinoma Cells

**DOI:** 10.1371/journal.pone.0137663

**Published:** 2015-09-11

**Authors:** Tamami Ukaji, Yinzhi Lin, Kouji Banno, Shoshiro Okada, Kazuo Umezawa

**Affiliations:** 1 Department of Molecular Target Medicine, Aichi Medical University School of Medicine, 1–1 Yazako-Karimata, Nagakute, 480–1195, Japan; 2 Department of Obstetrics and Gynecology, Keio University School of Medicine, 35 Shinano-machi, Shinjuku-ku, Tokyo, 160–8582, Japan; 3 Department of Pharmacology, Aichi Medical University School of Medicine, 1–1 Yazako-Karimata, Nagakute, 480–1195, Japan; Thomas Jefferson University, UNITED STATES

## Abstract

Previously we isolated migracin A from a *Streptomyces* culture filtrate as an inhibitor of cancer cell migration. In the present research, we found that migracin A inhibited migration and invasion of ovarian clear cell carcinoma ES-2 cells. In the course of our mechanistic study, migracin A was shown to enhance vasohibin-1 expression in an angiogenesis array. We also confirmed that it increased the mRNA expression of this protein. Moreover, overexpression of vasohibin-1 lowered the migration but not the invasion of ES-2 cells. Then, we looked for another target protein employing a motility array, and found that migracin A lowered the IGF-1 expression. Knockdown of IGF-1 by siRNA decreased the migration and invasion of ES-2 cells. Migracin A also decreased Akt phosphorylation involved in the downstream signaling. Crosstalk analysis indicated that overexpression of vasohibin-1 decreased the IGF-1 expression. On the other hand, it showed no direct anticancer activity in terms of the ES-2 growth in agar. Migracin A inhibited the migration and IGF-1 expression in not only ES-2 but also another ovarian clear cell carcinoma JHOC-5 cells. In addition, it also inhibited capillary tube formation of human umbilical vein endothelial cells. Since its cytotoxicity is very low, migracin A may be a candidate for an anti-metastasis agent not exhibiting prominent toxicity.

## Introduction

**Fig 1:**
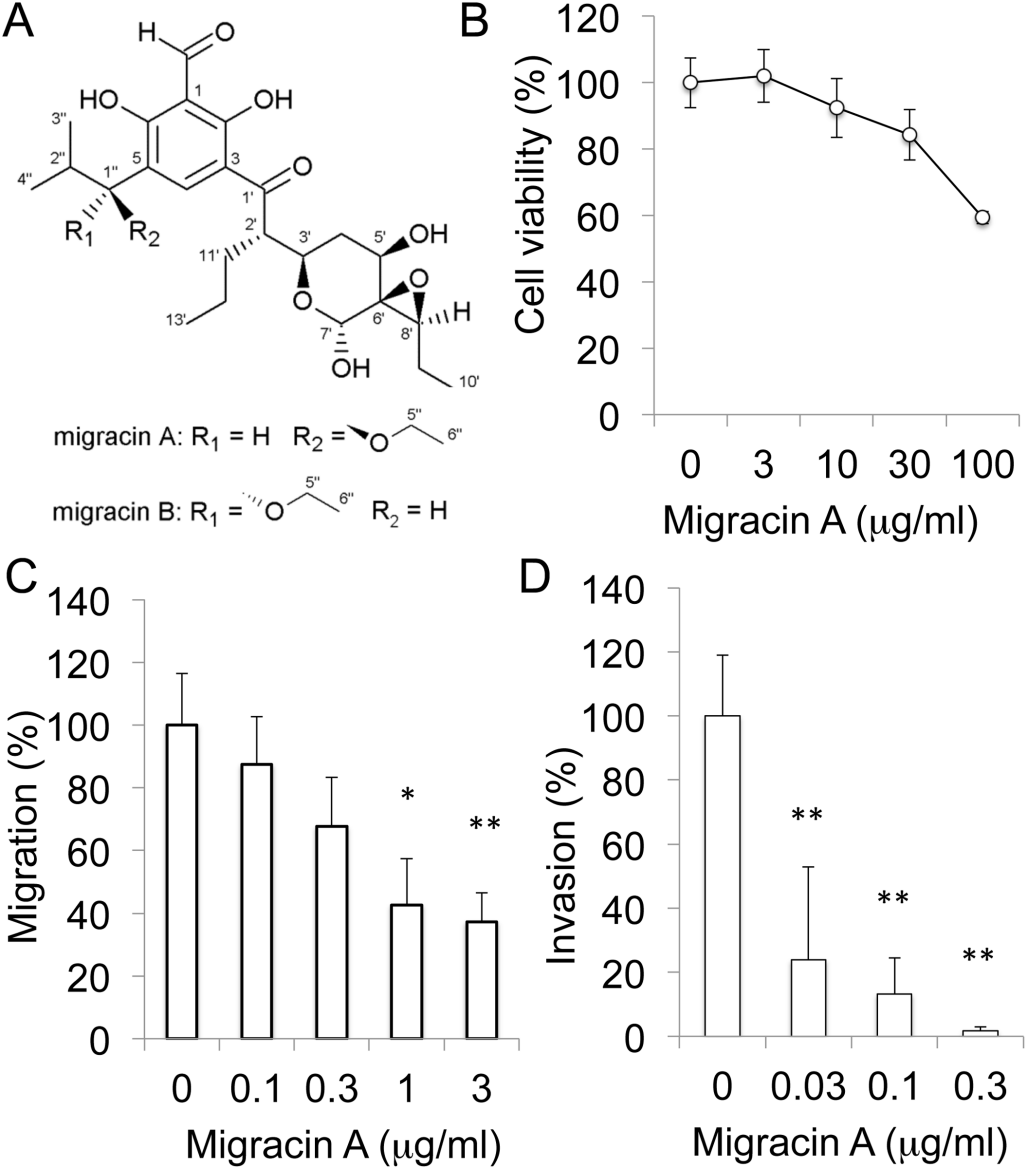
Inhibition of cellular migration and invasion by migracin A. A. Structures of migracin A and B. B. Effect of migracin A on the viability of ES-2 cells. The cells were incubated with migracin A for 24 h, and viability was assessed by MTT. C. Inhibition of cellular migration. Migration was measured by wound healing assay. The cells were incubated for 24 h. D. Inhibition of cellular invasion. Invasion was measured by Matrigel chamber assay. The cells were incubated for 24 h. *, P<0.05. **, P<0.01 (n = 3).

The process of cancer metastasis includes detachment from the primary tumor, migration, invasion, transport in the blood or lymphatic vessels, and attachment at the secondary site. Migration is especially involved in the mechanism of all types of cancer metastasis. Therefore, we looked for cellular migration inhibitors of low molecular weight from microbial culture filtrates. As a result, we discovered novel compounds, migracin A and B, from the culture filtrate of *Streptomyces* sp [1]. Migracin A and B inhibited cellular migration in human breast carcinoma MDA-MB-231, fibrosarcoma HT1080, and lung carcinoma A549 cells without showing any cytotoxicity. Migracin A and B are closely related in structure (Fig 1A), and show similar inhibitory activities. The structure of migracin is related to that of luminacin C. Luminacin C was isolated from *Streptomyces* sp. as an inhibitor of capillary tube formation in human umbilical vein endothelial cells (HUVEC) [2].

Ovarian cancer is the most common cause of gynecologic disease-related death, with a 5-year survival rate of about 30%. Ovarian carcinomas often metastasize to neighboring organs such as lung, liver and the peritoneal cavity through direct extension, migration, invasion, and lymphatic vessel transport [3]. About 70% of patients with ovarian cancer are diagnosed at an advanced stage when the ovarian cancer has metastasized because patients usually are asymptomatic at early stages [4]. Moreover, clear cell ovarian tumors are part of the epithelial tumor group of ovarian cancers, and these tumors may have a worse prognosis than other epithelial type-ovarian tumors [5]. Therefore, effective metastasis inhibitors with low toxicity should be useful for therapy against ovarian clear cell cancer.

In our previous study, migracin A and B inhibited cell migration in several cancer cell lines. However, its molecular mechanism remains to be elucidated. In the present research, we first evaluated the inhibitory activity of migracin A on the migration and invasion of ovarian clear cell carcinoma ES-2 cells. Then, we looked into the mechanism of inhibitory activity using a protein array. As a result, we found that an increase of vasohibin-1is involved in inhibition of migration, and that a decrease of IGF-1 and downstream signaling is involved in inhibition of migration.

## Materials and Methods

### Materials

Migracin A was isolated from *Streptomyces* sp. as reported previously [1]. Doxorubicin was purchased from Wako Pure Chemical Industries, Ltd. (Osaka, Japan). Recombinant Human VEGF_165_ was purchased from R&D Systems (Minneapolis, MN). IGF-1 receptor kinase inhibitor Linsitinib and PI3K/Akt inhibitor LY294002 were purchased from Chemie Tek (Indianapolis, IN) and Wako Pure Chemical Industries, respectively.

### Cell culture

Human ovarian clear cell carcinoma ES-2 (ATCC, Manassas, VA) and JHOC-5 (kindly provided by Dr. N. Yanaihara, Jikei University School of Medicine, Tokyo, Japan) cells were cultured in Dulbecco’s modified Eagle’s medium (DMEM) supplemented with 10% (v/v) fetal bovine serum and penicillin/streptomycin at 37°C in a humidified incubator with 5% CO_2_. Primary isolated human umbilical vein endothelial cells (HUVECs; Cell Applications, Inc., San Diego, CA) were cultured at 37°C in a humidified incubator with 5% CO_2_ in modified endothelial growth medium (Cell Applications, Inc.)

### Cell viability assays

Cell viability was evaluated by an MTT assay. Cell suspensions (100 μl) at a density of 1× 10^4^ cells per ml were plated in 96-well microtiter plates and incubated for 24 h. Then, the migracin A solutions at different concentrations were added to each well and further incubated for 24 h under the same conditions. MTT solution was added to each well and incubated for 2 h. Then, the older medium containing MTT was gently replaced by DMSO and pipetted to dissolve formazan crystals which formed. Absorbance was then determined on a Microplate Reader (Bio-Rad Laboratories, Inc., Hercules, CA) at 570 nm.

### Wound healing assay

Cells in 24-well plates were allowed to reach confluence before the surface was uniformly scratched across the center of each well with a pipette tip. The wells were then rinsed twice with serum-free media to remove floating cells and growth media, after which the cells were cultured in serum-free media for 24 h. The initial wounded area and movement of the cells into the scratched area were recorded. Experiments were performed in triplicate in three independent experiments.

### Cell invasion assay

ES-2 cells were suspended in 500 μl of serum-free medium containing migracin A or the DMSO and seeded into the upper chambers coated with BD Matrigel Basement Membrane Matrix (Corning Inc., Corning, NY). The lower chambers were filled with 750μl of medium containing 10% FBS and incubated for 24 h at 37°C in a humidified CO_2_ incubator. Then, after fixation of the invading cells, non-invading cells remaining on the upper surface were removed by wiping with a cotton swab. Invading cells attached to the underside were stained with Diff-Quick solution (Sysmex, Kobe, Japan), and counted.

### Target protein profiling

Cells were treated with migracin A and incubated for 24 h. The cell culture supernatants were diluted and incubated with the human angiogenesis array kit (R&D Systems, Minneapolis, MN) as per the manufacturer’s instructions. Array data were developed on LAS4000 (GE Healthcare UK Ltd, Buckinghamshire, England) following exposure to chemiluminescent reagents. Total RNA was extracted from ES-2 cells using RNeasy mini (Qiagen, Hilden, Germany) 8 h after treatment of migracin A. A PCR motility array was purchased from Qiagen, and used as described in the manufacturer’s instructions. Data analysis was carried out using the comparative Ct method.

### RNA isolation and semi-quantitative RT-PCR analysis

Total RNA was extracted from cultured cells using TRIzol reagent (Life Technologies, Carlsbad, CA). Reverse transcription was carried out at 37°C for 120 min with High-Capacity cDNA Reverse Transcription kit (Life Technologies). The prepared cDNA was used for PCR amplification with Taq DNA polymerase (Toyobo, Tokyo, Japan). The number of PCR cycles for each product was determined after confirmation of the efficacy of amplification and after having defined the linear primers used for semi-quantitative RT-PCR, the number of cycles, and the annealing temperature, as follows: vasohibin-1, 5’- ATG GAC CTG GCC AAG GAA AT -3’ (forward) and 5’- CAT CCT TCT TCC GGT CCT TG -3’ (reverse), 31 cycles, 58°C; exogenous-vasohibin-1, 5’- CCT AAC CCT CTC CTC GGT CTC GAT TCT ACG -3’ (forward) and 5’- CAT CCT TCT TCC GGT CCT TG -3’ (reverse), vasohibin-2, 5’- ACG TCT CAA AGA TGC TGA GG -3’ (forward) and 5’- TTC TCA CTT GGG TCG GAG AG -3’ (reverse), 37 cycles, 53°C; IGF-1, 5’- TGC TCT CAA CAT CTC CCA TC-3’ (forward) and 5’-GCC TCC TTA GAT CAC AGC TCC -3’ (reverse), 38 cycles, 58°C; and **β**-actin, 5’- CTT CTA CAA TGA GCT GCG TG-3’ (forward) and 5’- TCA TGA GGT AGT CAG TCA GG-3’ (reverse), 21 cycles, 58°C. PCR products were electrophoresed on 2% agarose gels, stained with ethidium bromide, and visualized with a UV illuminator.

### Plasmid construction and Establishment of vasohibin-1-overexpressing cell lines

The human vasohibin-1 gene was amplified from the cDNA of ES-2 cells, and was subcloned into pcDNA3.1/Zeo plasmid vector (Life Technologies). The cell lines stably expressing V5-tagged vasohibin-1 were established by transfecting the vectors into ES-2 cells using Lipofectamine LTX (Life Technologies) followed by Zeocin (Life Technologies) selection. The cells transfected with pcDNA3.1/Zeo were designated ES-2-empty.

### Western blot analysis

For detection of the protein level, cells were lysed with lysis buffer (50 mM Tris—HCl, pH 7.5, 150 mM NaCl, 0.1% (w/v) SDS, 1% (v/v) Triton X-100, 1% (w/v) sodium deoxycholate, proteinase inhibitor (Sigma-Aldrich Co., St Louis, MO), and PhosSTOP (Roche Applied Sciences, Indianapolis, IN)) and centrifuged at 14,000×g for 10 min. The protein concentration of the supernatants was determined. Aliquots of the cell lysates with 6×sample buffer (350 mM Tris-HCl, pH 6.8, 30% glycerol, 0.012% bromophenol blue, 6% SDS, and 30% 2-mercaptoethanol) were subsequently boiled for 3 min and electrophoresed on SDS-polyacrylamide gels. Proteins were transferred to PVDF membranes and immunoblotted with anti-V5 (Gene Tex Inc., Irvine, CA), anti-β-actin (Santa Cruz Biotechnology, Inc., Santa Cruz, CA), anti-phospho-Akt (Ser473) and anti-Akt (Cell Signaling Technology, Danvers, MA) antibodies. Detection was performed with enhanced chemiluminescent reagent (Merck KGaA, Darmstadt, Germany).

### Knockdown of IGF-1 by siRNA

siIGF-1 (sc-37193) and small interfering RNA-A as control (sc-37007) were purchased from Santa Cruz Biotechnology Inc. Transfection of cells with siRNAs was carried out using the Lipofectamine RNAiMax transfection reagent (Life technologies) according to the manufacturer's instruction. The efficiency of transfection was determined by mRNA expression.

### Soft agar colony formation

A soft agar colony formation assay was employed according to the manufacturer’s instructions using CytoSelect 96-well Cell Transformation Assay kit (Cell Biolabs, San Diego, CA). Each well contained 50 μl of 0.6% agar in a complete medium as the bottom and feeder layer, and 75μl of 0.4% agar in a complete medium with 1× 10^4^ cells as the top layer. After 6 days colonies were lysed with lysis buffer and detected with CyQuant GR dye for the quantification of anchorage-independent growth and the fluorescence was measured with a fluorometer using the 485/520 filter set SpectraMax M5 (Molecular Devices, Sunnyvale, CA).

### Capillary tube formation assay

An in vitro angiogenesis assay kit was employed according to the manufacturer’s instructions (Merck KGaA). HUVEC (2 × 10^4^ cells/well) were seeded onto the surface of 48-well cell culture plates pre-coated with polymerized ECMatrix and then incubated at 37°C for 4 h. The tube formation was observed under a phase-contrast microscope. The total tube length in four random view-fields per well was measured by Image J software and average value was calculated.

## Results

### Inhibition of cellular migration and invasion by migracin A

Migracin A (Fig 1A) showed no prominent toxicity below 30 μg/ml, as shown in Fig 1B. It inhibited migration of ES-2 cells at 1–3 μg/ml employing the wound healing assay in 24 h (Fig 1C). It also inhibited invasion of the cells at 0.03–0.3 μg/ml in Matrigel chamber assay in 24 h (Fig 1D). Migracin A inhibited the invasion at about a 10 times lower concentration than with the migration, which is likely due to the much lower number of cells in the Matrigel analysis. Thus, migracin A was found to inhibit both migration and invasion of ES-2 cells without toxicity.

### Involvement of vasohibin-1 in inhibition of cellular migration

**Fig 2:**
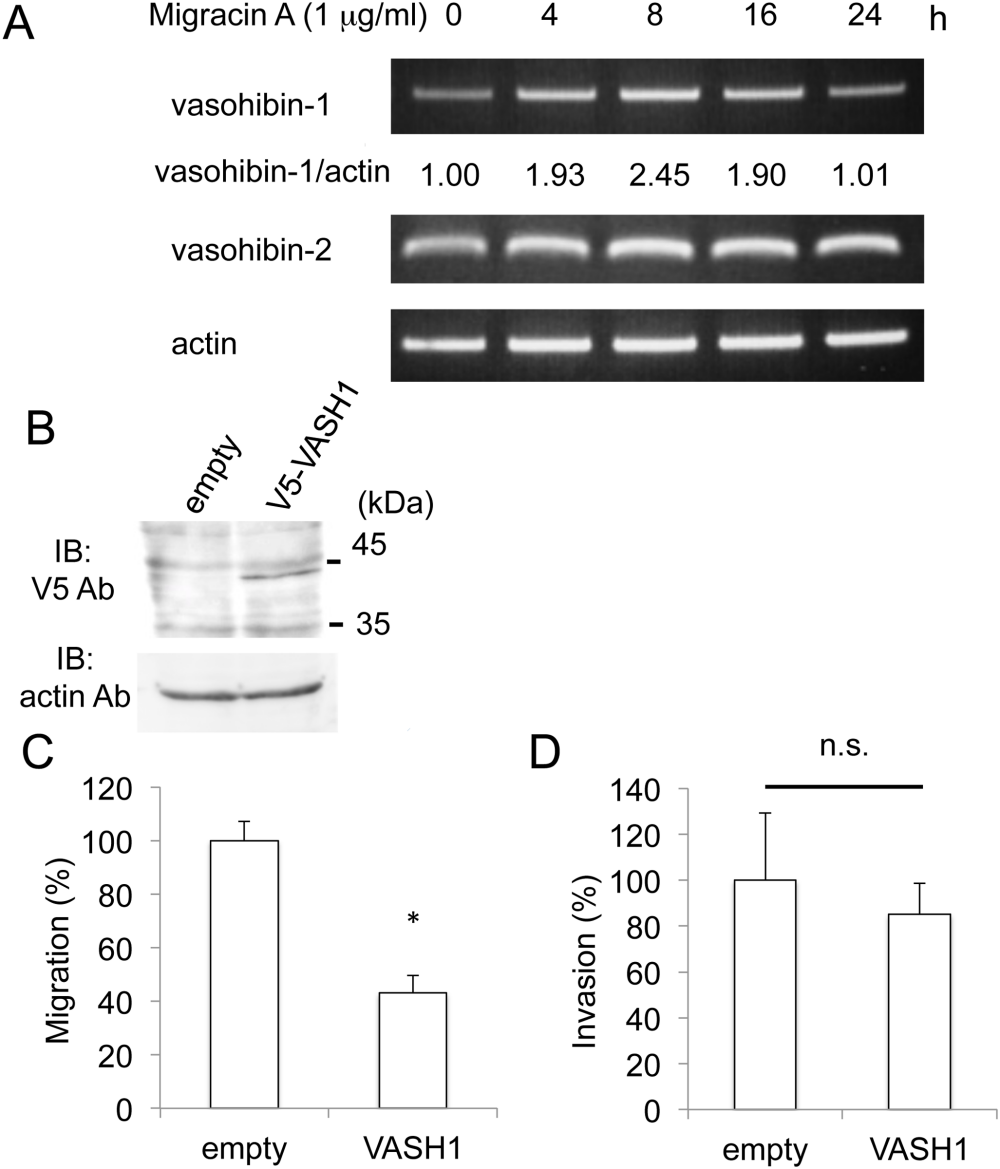
Involvement of vasohibin-1 in the inhibition of migration by migracin A. A. Effect of migracin A on vasohibin mRNA expressions in ES-2 cells. The cells were incubated with 1 μg/ml of migracin A, and each mRNA was measured by PCR. B. Over expression of V5-tagged vasohibin-1 in ES-2 cells. C. Inhibition of cellular migration by overexpression of vasohibin-1. D. Effect of vasohibin-1 overexpression on cellular invasion. *, P<0.01 (n = 3–6).

We employed a protein array for angiogenesis to study the mechanism of inhibition. ES-2 cells were treated with migracin A for 24 h, and protein expression was measured. Protein expression changed for some but not many, as shown in Table 1. Among these, we looked at vasohibin for further study, since its expression increased by about 3 times. Vasohibin is classified into -1 and -2, the former is inhibitory to angiogenesis and the latter promotional [6–8]. We confirmed that migracin A enhanced the vasohibin-1 mRNA expression, while not changing the vasohibin-2 expression (Fig 2A). We then prepared vasohibin-1-overexpressing ES-2 cells (Fig 2B), and the overexpression of vasohibin-1 was found to inhibit the migration (Fig 2C). However, cellular invasion was not changed by the vasohibin-1 overexpression (Fig 2D). Thus, up-regulation of vasohibin-1 is likely to be involved in the inhibition of migration.

**Table 1:** Protein array result for migracin A.

Protein	Expression level (% of control)
Endostatin	38.3
CXCL16	43.6
Coagulation Factor 3	55.7
Serpin B5	58.1
MIP-1 alpha	64.8
Vasohibin	316.8

Human angiogenesis array was used to detect proteins secreted from ES-2 cells into the culture medium. Cells were treated with migracin A and incubated for 24 h. The expression levels were calculated using the following formula: [expression level (%) = (protein level after treatment with migracin A − background value)/(protein level after treatment with DMSO − background value) × 100]. The top 6 proteins whose expression levels were changed by the treatment are listed.

### Involvement of IGF-1 in inhibition of cellular migration and invasion

**Fig 3:**
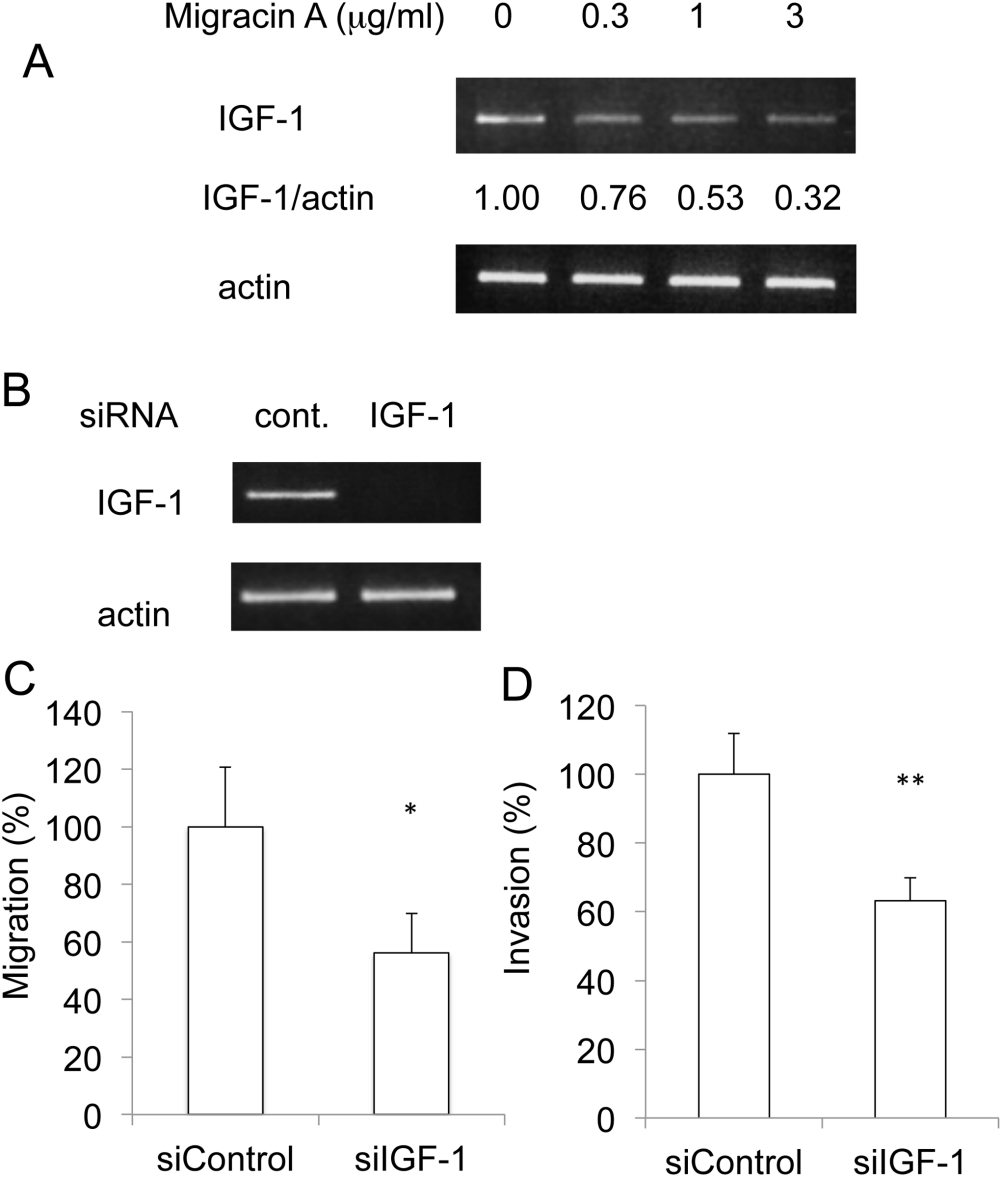
Involvement of IGF-1 in the inhibition of migration and invasion by migracin A. A. Inhibition of IGF-1 mRNA expression in ES-2 cells. The cells were incubated with indicated concentration of migracin A for 24 h, and the mRNA was measured by PCR. B. Knockdown of IGF-1 by siRNA in ES-2 cells. C. Inhibition of cellular migration by knockdown of IGF-1. D. Inhibition of cellular invasion by knockdown of IGF-1.

Since overexpression of vasohibin-1 did not inhibit the invasion, we next employed a PCR motility array for further mechanistic study (S1 Fig). Migracin A altered the expression of only a small number of proteins. Among them, migracin A inhibited the expression of IGF-1 that was reported to activate invasion [9–11]. We confirmed that migracin A lowered the mRNA expression in 24 h (Fig 3A). Knockdown of IGF-1 by siRNA (Fig 3B) was found to inhibit both the migration (Fig 3C) and invasion (Fig 3D) of ES-2 cells. Thus, down-regulation of IGF-1 is likely to be involved in the inhibition of migration and invasion.

### Inhibition of migration and invasion by IGF-1 receptor and PI3K/Akt inhibitors

**Fig 4:**
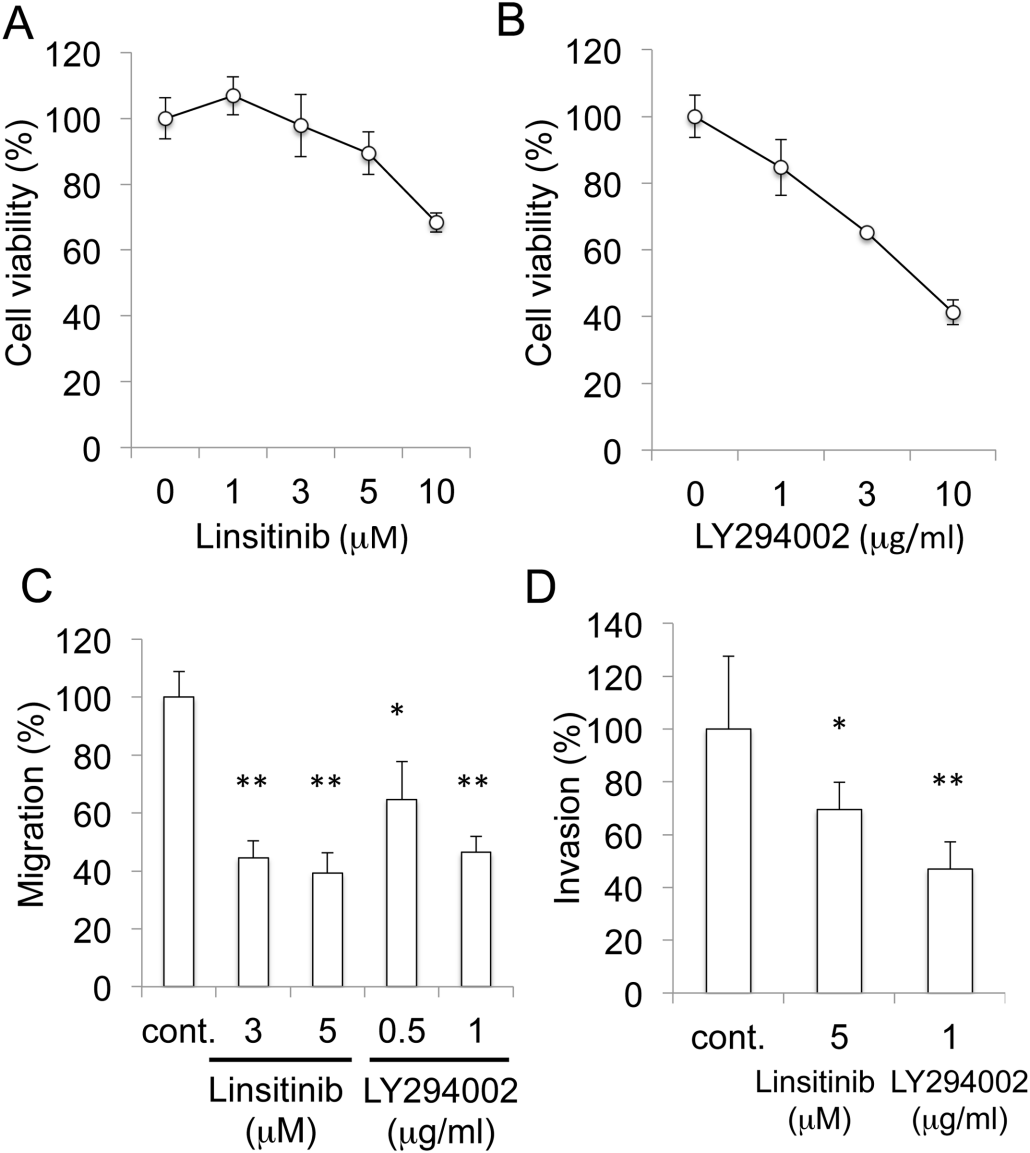
Inhibition of migration and invasion by IGF-1 receptor inhibitor Linsitinib and PI3K/Akt inhibitor LY294002. A. Effect of Linsitinib on the viability of ES-2 cells. The cells were incubated with Linsitinib for 24 h, and viability was assessed by MTT. B. Effect of LY294002 on the viability of ES-2 cells. The cells were incubated with LY294002 for 24 h, and viability was assessed by MTT. C. Inhibition of cellular migration. Migration was measured by wound healing assay. The cells were incubated for 24 h. *, P<0.05. **, P<0.01 (n = 3). D. Inhibition of cellular invasion. Invasion was measured by Matrigel chamber assay. The cells were incubated for 24 h. *, P<0.05. **, P<0.01 (n = 5).

We employed IGF-1 receptor inhibitor Linsitinib and PI3K/Akt inhibitor LY294002 to study the mechanism of inhibition. The cell viability was not markedly decreased with Linsitinib and LY294002 at 5 μM and 1 μg/ml, respectively (Fig 4A and 4B). Both Linsitinib and LY294002 inhibited the migration (Fig 4C) and invasion (Fig 4D) at these concentrations, respectively.

### Involvement of IGF-1/IGF receptor signaling in the effect of migracin

**Fig 5:**
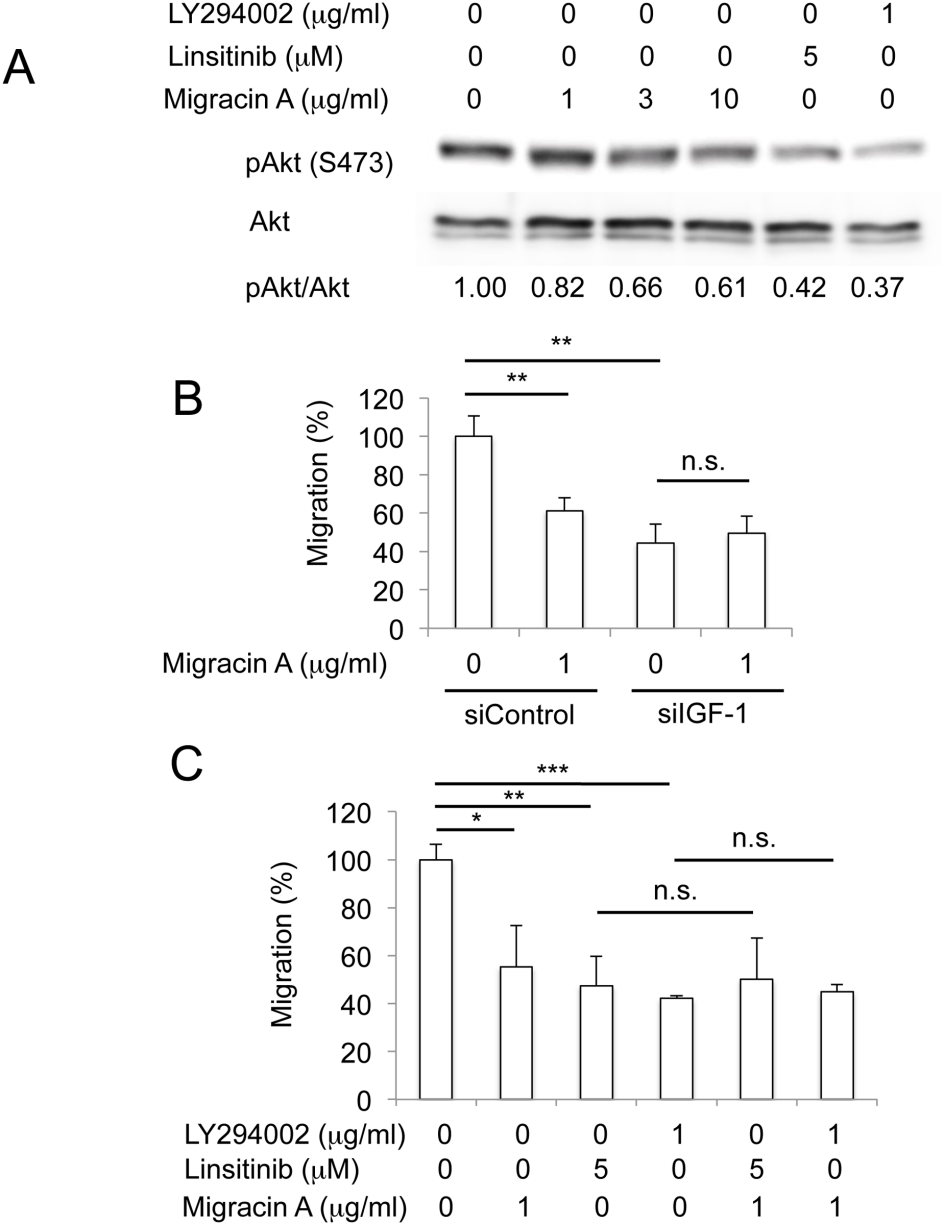
Involvement of IGF-1/IGF receptor signaling in the effect of migracin. A. Inhibition of Akt phosphorylation by migracin A. The cells were incubated for 24 h, and pAkt and Akt were detected by each antibody. B. Effect of migracin A on the migration in IGF-1-kockdown cells. The cells were incubated for 24 h. **, P<0.01. (n = 3) C. Effect of migracin A on the migration in Linsitinib or LY294002-treated cells. *, P<0.05. **, P<0.01. ***, P<0.001 (n = 3).

Akt phosphorylation is involved in the IGF-1/IGF-1 receptor signaling pathway. Migracin was shown to decrease Akt phosphorylation as IGF-1 receptor inhibitor Linsitinib and PI3K/Akt inhibitor LY294002, as shown in Fig 5A. Moreover, migracin failed to further inhibit migration in IGF-1-knokdown cells, as shown in Fig 5B. Migracin also did not lower the migration in Linsitinib or LY294002-treated cells (Fig 5C). Thus, inhibition of IGF-1/IGF-1 receptor signaling by migracin A is likely to be the essential mechanism of inhibition of migration.

### Decrease of IGF-1 expression by vasohibin-1 over expression

**Fig 6:**
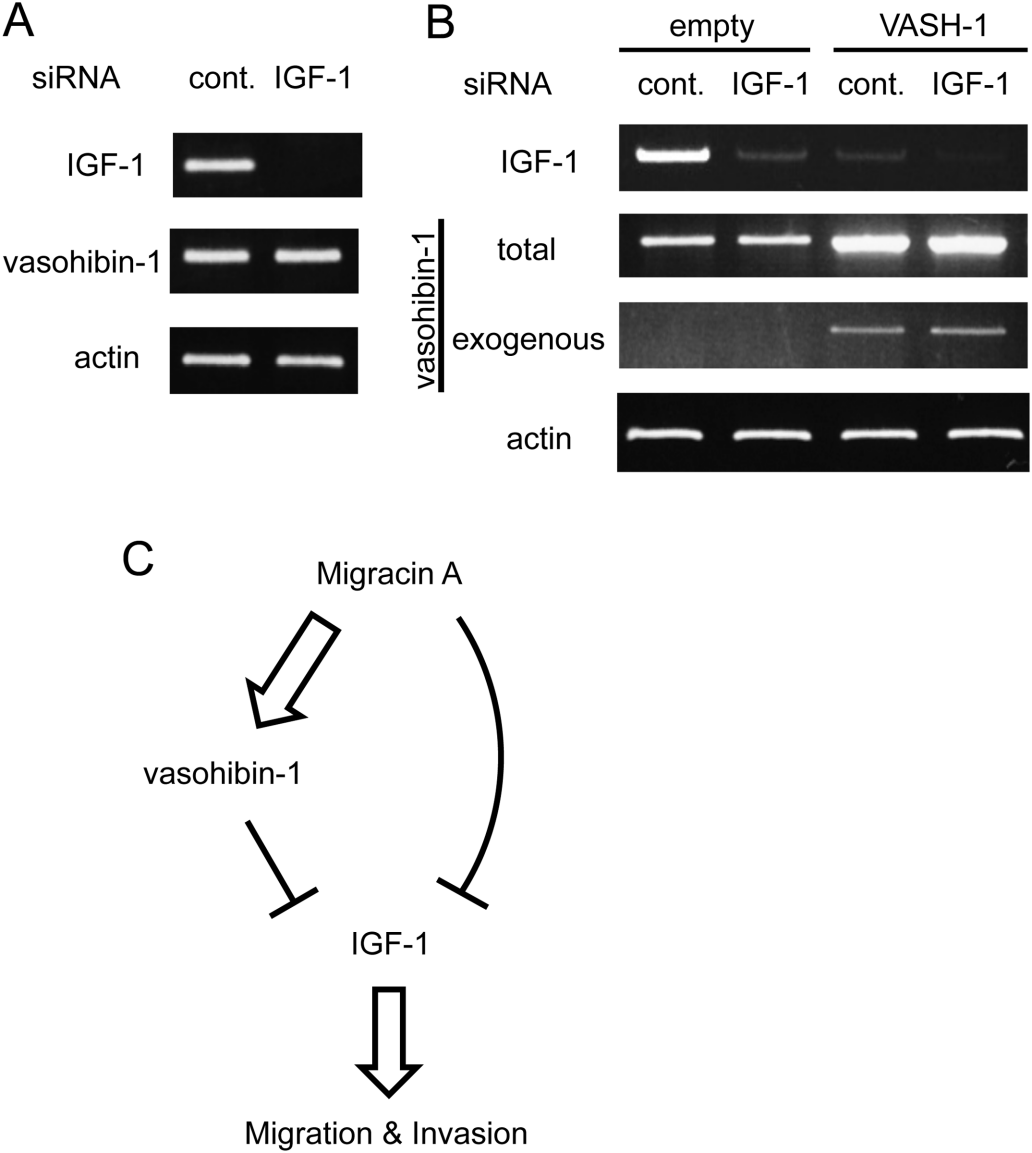
Relation between vasohibin-1 and IGF-1 expressions. A. Effect of IGF-1 knockdown on vasohibin-1 expression. B. Decrease of IGF-1 expression by the overexpression of vasohibin-1. C. Possible mechanism for inhibition of migration and invasion by migracin A.

We then studied the relation between vasohibin-1 and IGF-1 expressions. Knockdown of IGF-1 did not change the vasohibin-1 expression (Fig 6A). However, interestingly, overexpression of vasohibin-1 significantly decreased the IGF-1 expression (Fig 6B). A possible mechanism for inhibition of migration and invasion by migracin A is illustrated in Fig 6C.

### Effect of migracin A on soft agar colony formation

**Fig 7:**
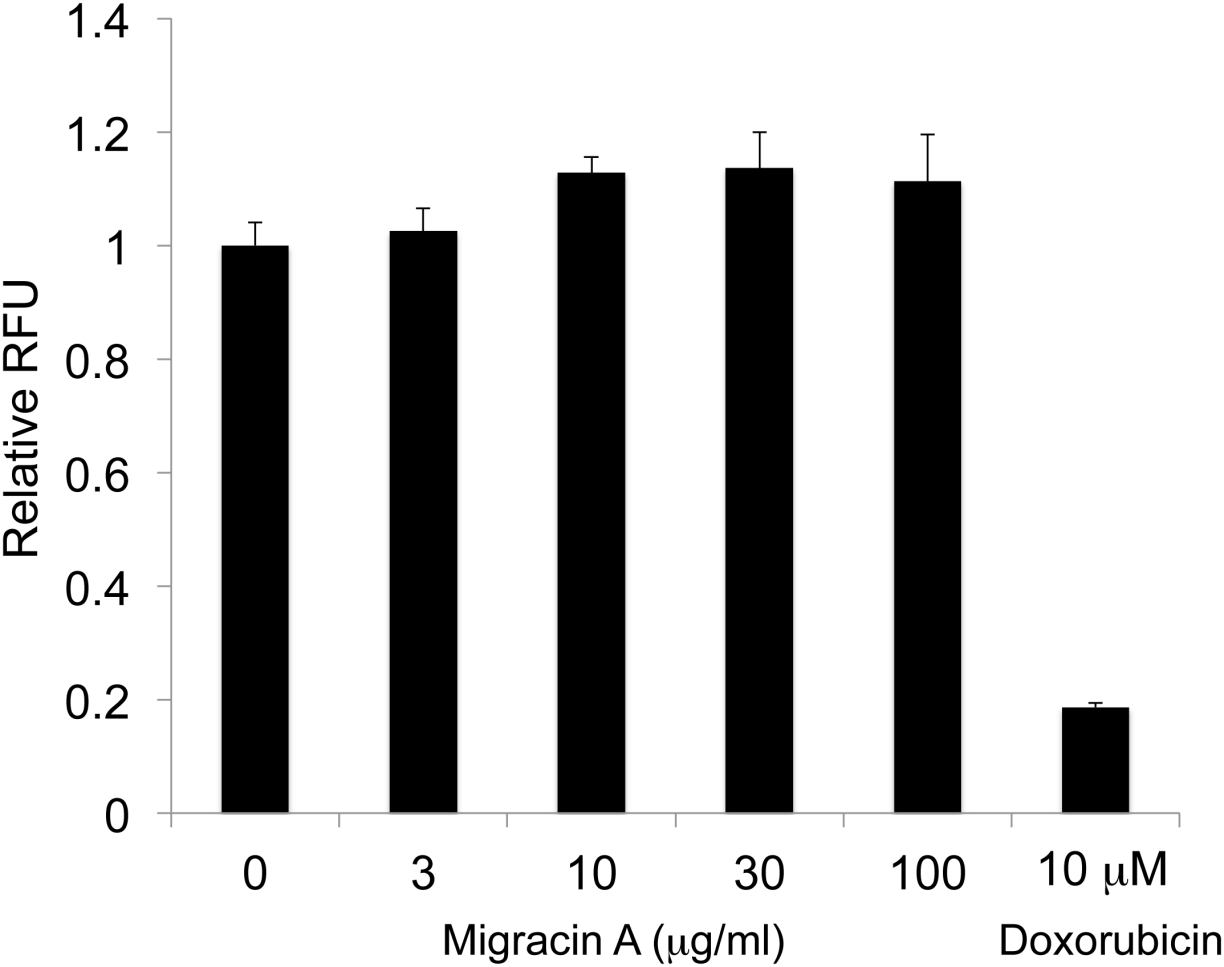
Effect of migracin A on soft agar colony formation of ES-2 cells. The cells were incubated for 6 days with each chemical. *, P<0.001.

We evaluated the direct anticancer effect of migracin A employing a colony formation assay in soft agar. As shown in Fig 7, it did not inhibit the colony formation even at 100 μg/ml (about 0.25 mM), while doxorubicin was effective at 10 μM.

### Inhibition of migration and IGF-1 expression by migracin A in ovarian clear cell carcinoma JHOC-5 cells

**Fig 8:**
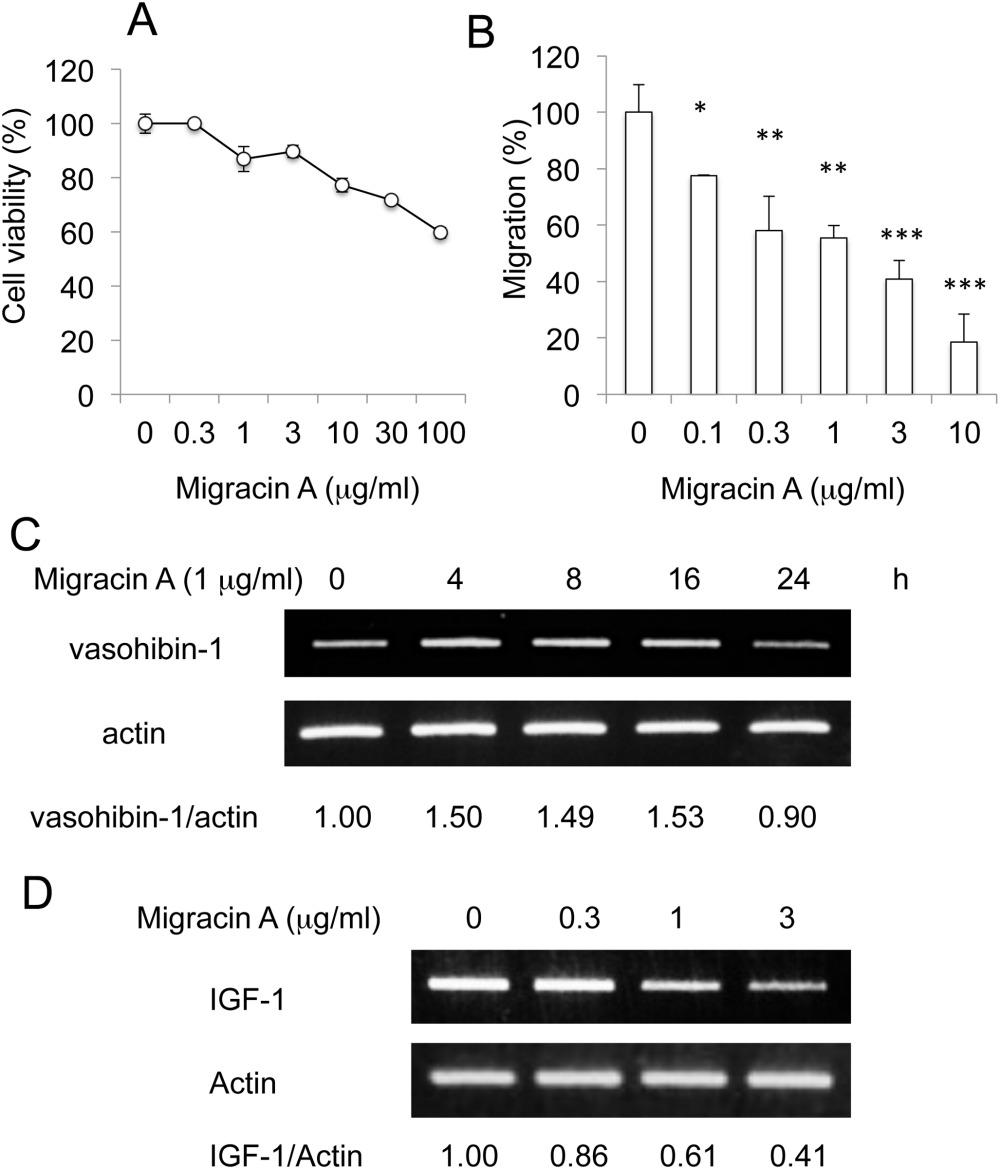
Inhibition of migration and IGF-1 expression by migracin A in JHOC-5 cells. A. Effect on viability. The cells were incubated with migracin A for 24 h. B. Inhibition of migration in JHOC-5 cells. The cells were incubated for 24 h. *, P<0.05. **, P<0.01. ***, P<0.001 (n = 3). C. Increase of vasohibin-1. The cells were incubated with 1 μg/ml of migracin A, and each mRNA was measured by PCR. D. Inhibition of IGF-1 mRNA expression. The cells were incubated with indicated concentration of migracin A for 24 h, and the mRNA was measured by PCR.

We employed human ovarian clear cell carcinoma JHOC-5 cells in addition to ES-2 cells. Migracin A inhibited the migration at the concentrations without prominent toxicity (Fig 8A and 8B). It increased the expression of vasohibin-1 (Fig 8C) and decreased the IGF-1 expression (Fig 8D). Thus, migracin A was active to inhibit migration in not only ES-2 cells but also JHOC-5 cells.

### Inhibition of capillary tube formation

**Fig 9:**
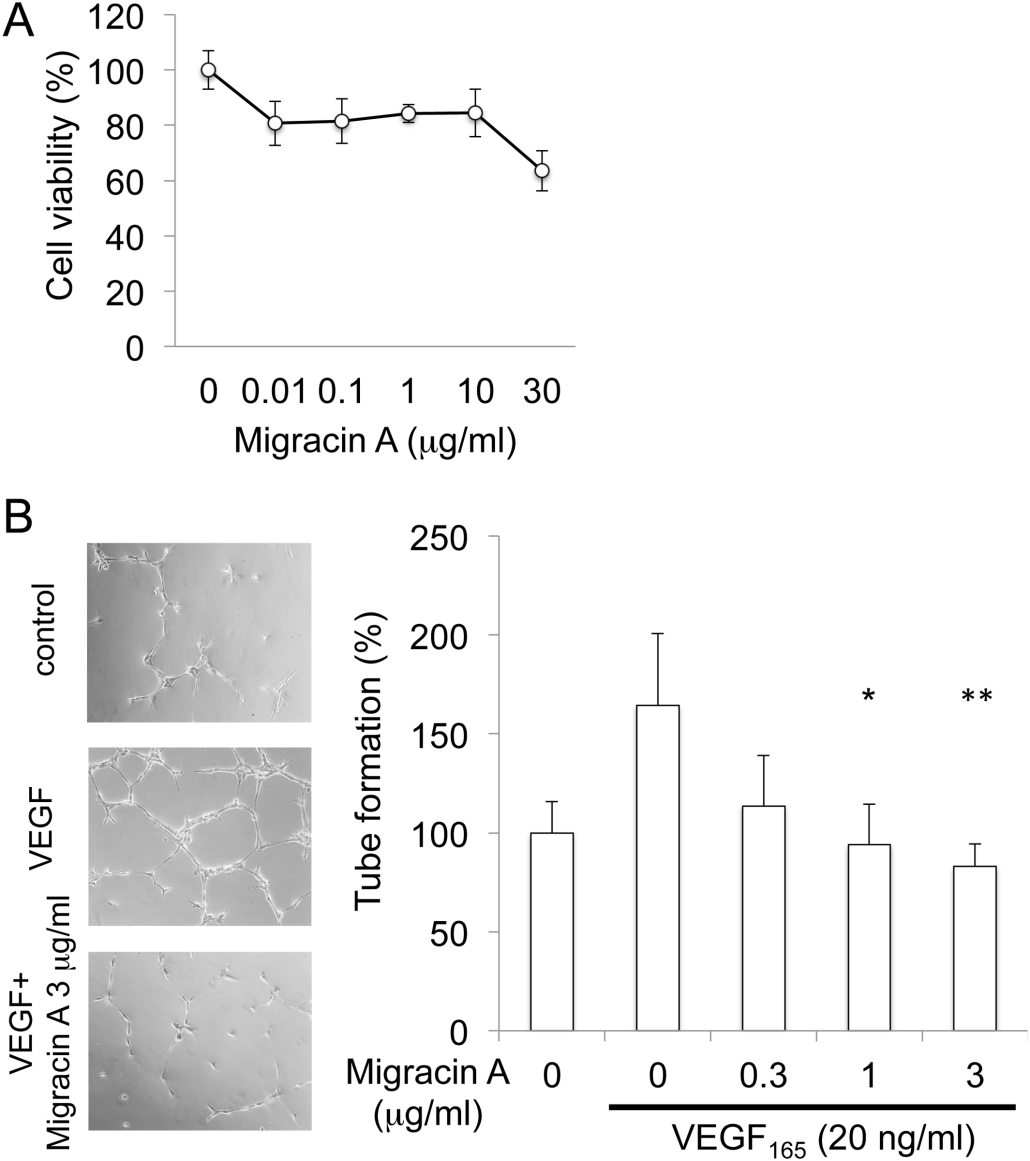
Inhibition of capillary tube formation by migracin A. A. Effect of migracin A on viability of HUVEC. The cells were incubated for 24 h, and viability was assessed by MTT. B. Inhibition of VEGF-induced tube formation by migracin A in HUVEC. The cells were incubated for 4 h. *, P<0.05. **, P<0.01 (n = 4).

Migracin A showed no prominent toxicity at 10 μg/ml (Fig 9A) on HUVEC, and inhibited VEGF-induced tube formation at 1–3 μg/ml, as shown in Fig 9B. Thus, migracin A shows anti-angiogenesis activity in addition to the inhibition of cancer cell migration and invasion.

## Discussion

Migracin A inhibits migration and invasion of ES-2 cells through the alteration of vasohibin-1 and IGF-1 expressions. It inhibited the migration, increased the vasohibin-1 expression, and decreased the IGF-1 expression in not only ES-2 cells but also in another ovarian clear cell carcinoma JHOC-5 cells (Fig 8).

Microorganisms produce a variety of small molecular compounds, and we isolated the novel compounds, migracin A and B, from *Streptomyces* as inhibitors of cancer cell migration. Luminacin C, having a related structure, was also isolated from *Streptomyces* as an inhibitor of capillary tube formation in HUVEC [2, 12]. Racemic luminacin C, called UCS15A, was reported to inhibit bone resorption [13] and cellular invasion [14]. UCS15A was also reported to inhibit Src homology-3 (SH3)-mediated protein-protein interactions resulting in a decrease in MEK activity [15,16]. We studied the effect of migracin A on MEK, but it did not affect the MEK activity (data not shown), suggesting that the mechanism of inhibition is different.

In the present research, we found that migracin A enhanced the vasohibin-1 expression, and its overexpression reduced the cellular migration. Vasohibin-1 was isolated as a negative-feedback regulator of angiogenesis, and its expression is increased in endothelial cells by proangiogenic stimulatory growth factors, such as VEGF and FGF-2 [17]. Then, a homologous gene named vasohibin-2 was identified [18]. Transfection of vasohibin-1 gene to lung carcinoma cells inhibited tumor angiogenesis and tumor growth in animal models [17]. Knockdown of vasohibin-1 increased neovascularization in a mouse ischemic retinopathy model. Conversely, intraocular injection of recombinant vasohibin-1, or an adenoviral vector containing a vasohibin-1 expression cassette, strongly suppressed retinal neovascularization in this model [19]. And further, transfection of the vasohibin-1 gene could attenuate bleomycin-induced pulmonary fibrosis via inhibition of angiogenesis [20].

Since vasohibin-1 overexpression did not inhibit cellular invasion, we continued screening of the mediator proteins, and found that IGF-1 expression was inhibited by migracin A. IGF-1 is known to drive tumor cell motility as an upstream effector of the PI3K/Akt signaling pathway, in that it activates cell proliferation, survival, migration and angiogenesis in several cancer cells [21]. Moreover, IGF-1 induced cellular migration and invasion in colon carcinoma cells [9, 22]. In ovarian cancer, an elevated serum level of IGF-1 is often observed [23]. High levels of IGF-1 are also found to be associated with increased disease risk, tumor metastasis and a poor prognosis in ovarian cancer [24]. Migracin A decreased phosphorylated Akt, similarly with an Akt inhibitor or an IGF-1 receptor antagonist (Fig 5A). Moreover, migracin A did not further inhibit the migration in siIGF-1-treated (Fig 5B) and the LY294002 or Linstinib-treated (Fig 5C) cells. Therefore, inhibition of IGF-1 signaling is considered to be the major cause of inhibition.

Since vasohibin-1 overexpression decreased the IGF-1 expression in our study, vasohibin-1 may partly down-regulate IGF-1. However, this signaling should not be enough to explain the inhibition of cellular invasion.

Epithelial-mesenchymal transition (EMT) is known to activate migration and invasion. We have tried to study the effect of migracin A on EMT. ES-2 cells are reported to possess a mesenchymal character [25], and we confirmed the expression of Twist in this cell line by PCR. Then, we studied whether migracin A would decrease the expression of Twist. However, migracin A did not decrease the twist expression by PCR. Therefore, it is considered that migracin A would not alter the mesenchymal character of ES-2 cells.

In conclusion, migracin A inhibited migration and invasion in ovarian clear cell carcinoma cells. An increase of vasohibin-1 and a decrease of IGF-1 are likely to be involved in the mechanism. In addition, migracin A may be a candidate for an anti-metastasis agent with low toxicity.

## Supporting Information

S1 Fig Results of PCR array analysis with migracin A in ES-2 cells. IGF-1 is shown by black triangle.
(TIF)
