# Supplementary figures and images for: Inhibition of IGF-1-Mediated Cellular Migration and Invasion by Migracin A in Ovarian Clear Cell Carcinoma Cells
Source: PLoS One. 2015 Sep 11;10(9):e0137663. doi: 10.1371/journal.pone.0137663 (PMC4567285; doi:10.1371/journal.pone.0137663)

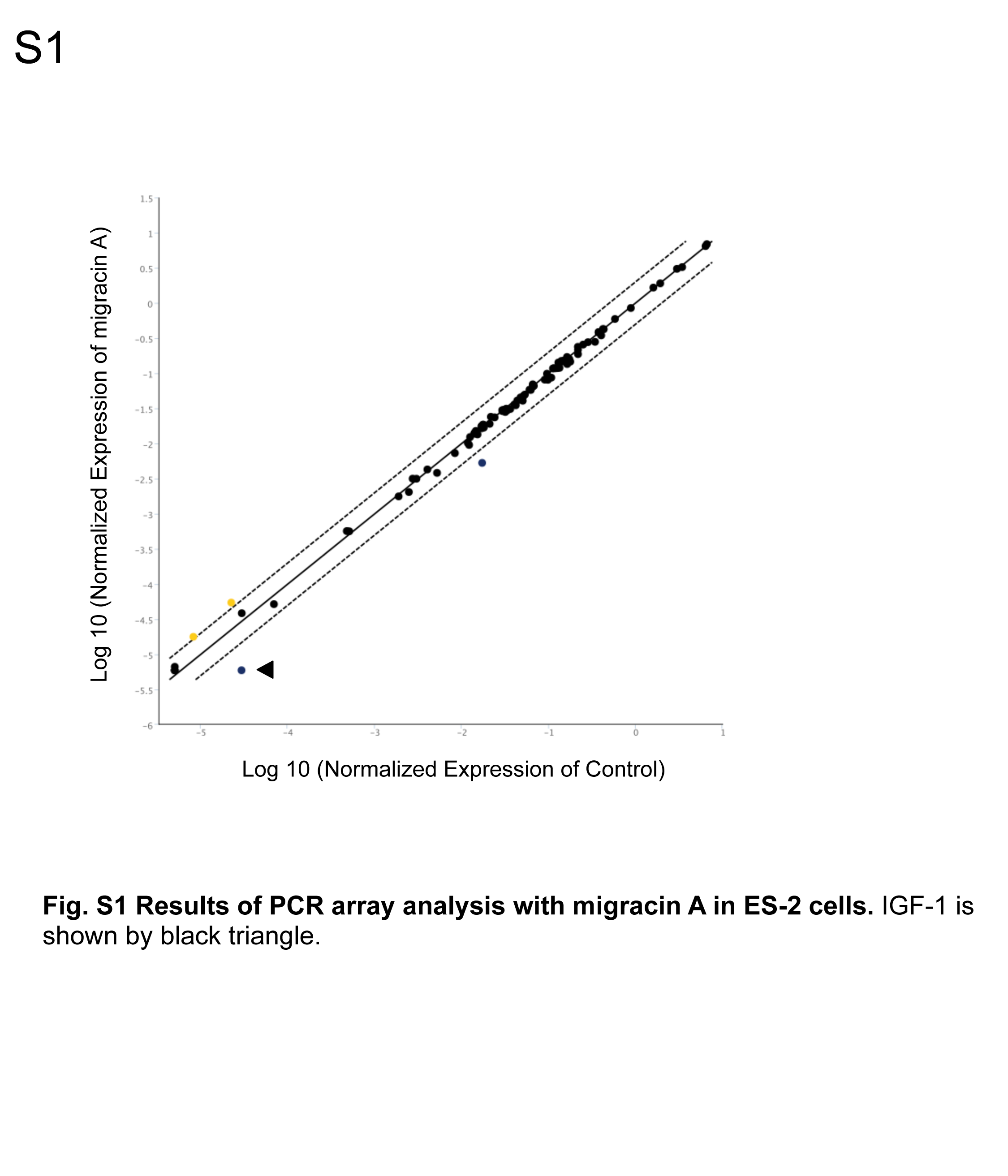

Supplement: S1 Fig — IGF-1 is shown by black triangle. (TIF) [file pone.0137663.s001.tif]
